# The use of ICT by ESL teachers working with young learners during the early COVID-19 pandemic in Poland

**DOI:** 10.1007/s10639-021-10556-6

**Published:** 2021-04-29

**Authors:** Paulina Marchlik, Kamila Wichrowska, Ewelina Zubala

**Affiliations:** Faculty of Education, University of Warsaw, Warsaw, Poland

**Keywords:** ICT, Young learners, COVID-19, ESL teachers, Remote education, Educational institutions

## Abstract

The article presents selected aspects of the empirical qualitative research conducted by the authors at the end of the first wave of the COVID-19 pandemic in Poland, in July and August 2020. As in March 2020 SARS-CoV-2 virus started to spread, the Polish Ministry of Education decided to close nearly all educational institutions, and teachers were forced to carry out lessons using the means of distance education. The authors of this paper tried to establish how teachers of English as a second language (ESL) working with young learners in three different types of institutions (public and private (pre-primary and primary) schools, language schools) coped with the situation of teaching a foreign language under these unusual circumstances. One of the results showed that teachers in three types of settings received different support. This either helped them to cope with the new situation of remote teaching, e.g. by choosing a platform for online teaching or providing meetings with a methodologist (private settings and language schools) or caused more confusion by leaving the decisions to the teachers (public settings). The findings of the study may contribute to the knowledge of remote education development and implementation of new technologies in teaching English to young learners, which may result in better quality language education in the future.

## Introduction

At the beginning of 2020 most countries all around the world started to suffer from a new and unpredictable virus SARS-CoV-2 that causes a disease known as COVID-19. Poland too, was affected by the virus, and during the first wave of the pandemic, the functioning of all institutions was limited with only a few days’ notice. The tasks of educational institutions needed to start being performed in a different form, which no teachers or learners were prepared for, i.e., by means of remote education and online teaching. The suspension of face-to-face classes in most educational facilities in Poland (including schools, preschools, language schools) took place on 12th March 2020, following the WHO announcement of the outbreak of a global pandemic. At that time the Polish Ministry of Education informed that the classes would only be temporarily suspended, until 25th March 2020 (Journal of Laws, 2020 item 410), but as it occurred later, that was just the beginning of a much longer period of remote education. From 25th March, preschools, schools and other educational entities were obliged to continue their work with the means of remote education. Teachers were left with limited to no help from the government, and the constant lack of valid information about when they would be able to return to in-class teaching caused additional confusion and anxiety. Apart from living in constant disinformation, everyone experienced a growing feeling of insecurity, the threat of being exposed to the coronavirus, reduction of salaries, and working from home while coping with additional family responsibilities, such as helping own children with distance learning (United Nations, 2020). On 25th May 2020 some teachers and students (e.g. primary schools: first to third graders) were able to return to school settings and have regular classes (Journal of Laws, 2020 item 780, 871). With the beginning of September 2020 all schools and preschools started a new school year in the buildings, only to go back to distance education on 9th November 2020 (the second schools closure concerned all students but preschool children and 6-year-old students, who in the Polish system attend a *reception class*).

In this article the authors take a closer look at how teachers of English as a second language (ESL) working with young learners in Poland coped with the unexpected transition from face-to-face classes to distance (online) teaching and what tools they used for this purpose during the first wave of the COVID-19 pandemic. We present a unique, qualitative look at the problems caused by implementing the first remote learning solutions in three different school environments: public, private (pre-primary and primary schools) and language schools. It seems that the differences resulting from this division have not been previously recognised.

## Theoretical and empirical foundations

The unexpected transition from face-to-face classes to distance education in early spring of 2020 was experienced by teachers all over the globe. Literature review shows a variety of terms used to describe what happened in the world of education with the closure of educational institutions following the announcement of the COVID-19 pandemic. Authors talk about mandated online schooling (Judd et al., 2020); emergency eLearning (Murphy, 2020), emergency remote teaching (Bozkurt & Sharma, 2020; Hodges et al., 2020), emergency online teaching (Kirschner & Neelen, 2020; Şener et al., 2020), emergency remote online teaching (Jeffery & Bauer, 2020), ‘forced’ transition to remote teaching (Carrillo & Flores, 2020), ‘sudden transformation of education to the new on-line distant technological teaching environment’ (Kitishat et al., 2020).

One thing is certain – the transition was sudden and unexpected in many countries, and Poland was not an exception. The rapid switch to distance teaching was a source of uncertainty and anxiety for both students and teachers, as neither the learners nor the educators had prior experience with this kind of education (85% of Polish teachers had no experience with online teaching; see: Buchner et al., 2020). The situation simply came as a surprise to everyone, and people needed time to adjust to the new online-based educational reality.

While over a half (51% in 2019) of the world’s population have access to the Internet (ITU, 2021), slightly over 90% of Polish households have Internet connection at home (GUS, 2020a; OECD, 2021), which means that the percentage of Internet connections in Poland is above the world average. At the same time, in Poland 99.5% households with children have broadband Internet connection (GUS, 2020b), while approximately only 60% of Eastern European children and young people aged 25 years or younger have Internet access at home (UNICEF & ITU, 2020).

According to Blurton (1999), information and communication technologies (ICT) are a diverse set of technological tools and resources used to communicate, as well as to create, store, disseminate, and manage information. It is worth pointing out that ICT are not only a useful communication tool, they are also used to expand the range of teaching tools, such as: interactive boards, computers, tablets, online platforms with ready to use materials, etc. The Internet allows the teachers to communicate with students in both synchronous and asynchronous ways, thus facilitating e-learning (Hrastinski, 2008). Asynchronous communication tools, such as email or learning platforms, allow the learners to study at their own pace and at the time most convenient for them, while synchronous communication tools, such as video conferencing platforms, facilitate real-time interaction between the teacher and the students (Ally, 2004).

A model of the remote education process proposed by Plebańska et al. (2020) implies the use of different tools to achieve different educational goals. Plebańska suggests working with: multimedia resources to introduce the material, interactive activities to consolidate the content and quizzes and review tasks to verify the results. It is also recommended to use individual or group projects, followed by students’ own work using traditional educational resources. On top of that the teachers should plan synchronous contact with students, creating an opportunity not only to conduct a substantive meeting in real time, but also, and perhaps most importantly, to talk, discuss current challenges and problems. Such contact builds relationships and unites the group (Plebańska, 2020).

Nevertheless, it would be wrong to assume that both synchronous and asynchronous online education are suitable for all learners. While older students who are independent learners can benefit from online distance education, as they are able to control their learning at their own pace, younger children are dependent learners and need more guided instruction (Di Pietro et al., 2020), and in their case asynchronous learning requires assistance of the parents/guardians.

As far as teaching young learners is concerned, it should be highlighted that young children need a lot of fun, play and movement. Frequent change of activities and forms of work is a necessity, as they have short attention span. Language teachers should provide children with a variety of interactions – with the teacher, with other students and with didactic aids, such as: real objects, puppets, worksheets, toys, etc. The key to an educational success in teaching English to young learners (TEYL) is learning by doing. To make the language experience meaningful for children, it should take place in a specific situational context (Shin & Crandall, 2013). Therefore, when it comes to remote education, it seems crucial to provide children with a lot of interactive activities (Plebańska, 2020) to use “the potency of play” (Ostroff, 2020).

In Poland, during the first schools closure, the tasks of educational institutions could be carried out using distance education methods and techniques, but the term “distance education” was not specified. In practice, this meant reaching for remote education tools to deliver the curriculum, although this was often asynchronous teaching, i.e. teachers sent the material and instructions to students and, in the case of young learners, their parents. Interestingly, young learners are the largest group of students (41%) who did not have synchronous classes in real time (Librus, 2020).

It was all the participants in the education process who were affected by the situation; since distance education was not a choice but a necessity (Pyżalski, 2020), it was not only teachers and students, but also parents who had to face the challenge of remote classes (Karolczuk, 2020). Almost one fifth of parents indicated no school’s effort to start providing real-time online lessons in the first months of school closure (Librus, 2020). In addition, the fixed lesson schedule used before the lockdown was no longer valid. Since, with the aim of reducing children’s screen time, online classes took place only once a week and often lasted no longer than 30 minutes (Journal of Laws, 2020 item 493), parents practically became their own children’s private teachers.

Taking into consideration all of the above facts, the authors of this article decided to explore the situation of ESL teachers and their ways of coping with foreign language education during the first wave of the coronavirus pandemic.

## The study

**Fig. 1:**
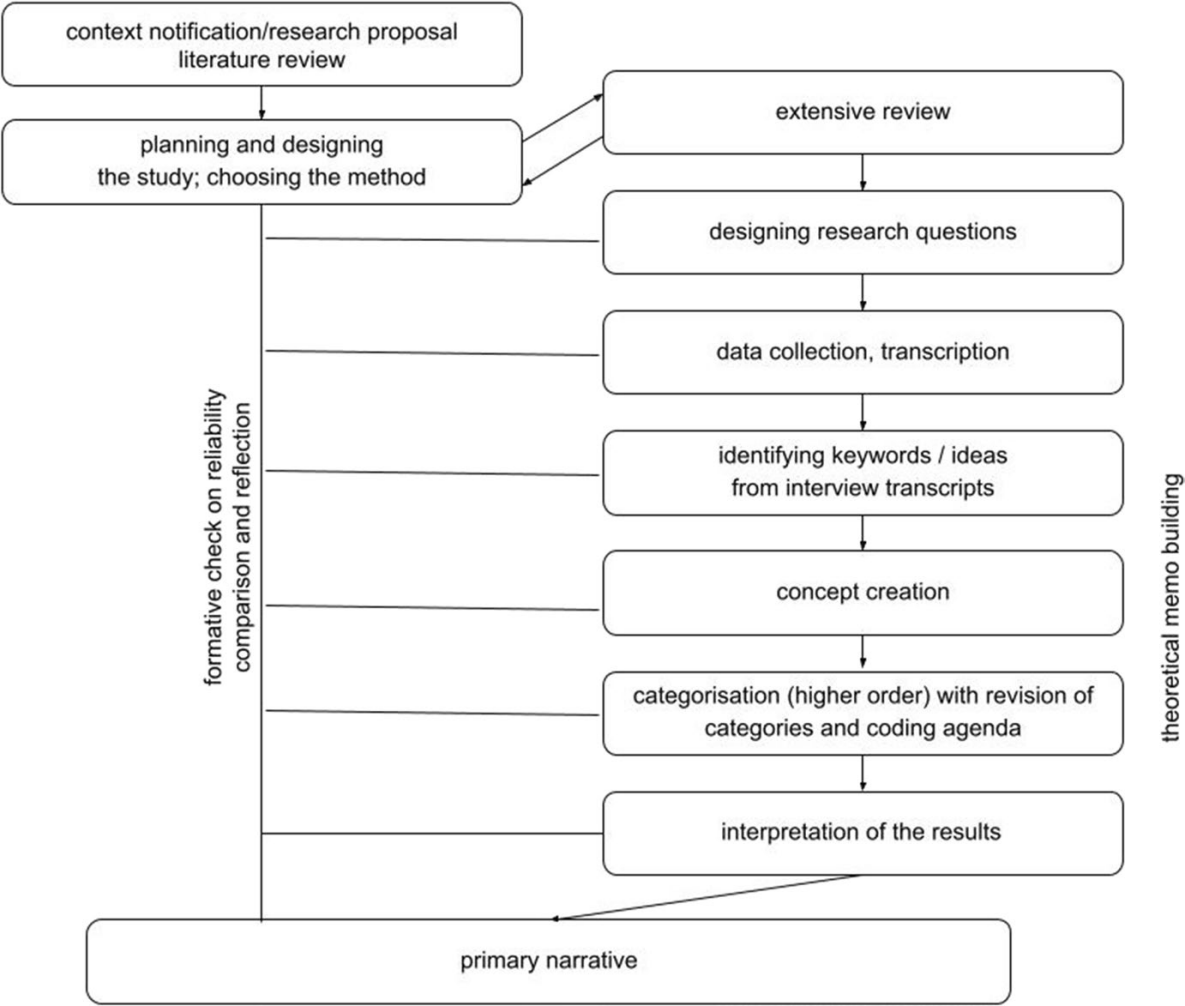
The research model applied (source: authors’ own presentation)

The study concerned the use of ICT and distance teaching experiences of teachers of English as a second language working with pre-primary and lower primary aged children in three different types of settings: (1) public (preschools and primary schools), (2) private (preschools and primary schools) and (3) language schools. The authors sought to answer the main research question, “Is there any distinction in the way teachers working in three different types of settings cope with (the unusual situation of) distance teaching forced by the spread of COVID-19?” The details of the research process are presented in the graph below (Fig. 1).

The study comprised interviews with twelve ESL teachers working with the youngest pupils in public and non-public kindergartens and primary schools, as well as in private language schools, located in villages, small and large towns. The participants in the study are teachers aged 25–50, with different formal qualifications and degrees of professional advancement. The survey was conducted in July and August 2020, after the end of the 2019/2020 school year and after the first lockdown during the first wave of the COVID-19 pandemic. All teachers participating in the study had experience in remote teaching during the first coronavirus-induced school closure, in either synchronous (real-time) or asynchronous form.

The selection of participants for the study was based on convenience, i.e., the teachers participating in the research were within a group that was easily available and therefore easy to contact (convenience sampling) (Saumure & Given, 2008). The persons who took part in the study were teachers that responded to the researchers’ invitation positively, and agreed for the interview to be recorded. All the study participants were informed that they may withdraw their consent at any time.

The study can be characterised as exploratory and descriptive. The researchers used the narrative interview (followed by semi-structured interview, for clarification) with the aim of getting the most information on the emergency remote teaching experiences of the ESL teachers, who were interviewed taking into account the restrictions of social distancing. The interviews were conducted fully online via Zoom.

Narrative interview is one of the qualitative methods used in social research, mainly in biographical studies, but also in studies involving significant events in individuals’ lives, but not encompassing their whole lives – the pandemic experience of lockdown and emergency remote teaching can without doubt be considered such an event. In narrative interviews, participants are asked to share their story and it is the narrator who makes decisions about events, their order and significance. Telling the story, the narrator constructs his/her own perception of reality (Kartch, 2017).

For data collection, we used the interview format systematised by Fritz Schütze (1983), in which the narrative interview has five phases: (1) beginning the interview, (2) asking an introductory question, (3) the narrator telling the story without any interruptions until he/she indicates the end of the story (the so-called coda), (4) asking clarifying questions by the researcher, and (5) ending the interview (Jovchelovitch & Bauer, 2000; Szczepanik & Siebert, 2016).

Each interview started with an initial generative question: “Could you tell me about your experiences with remote education during the pandemic?” The informant then presented his or her narrative, uninterrupted by additional questions. Only when the narrator indicated the end of their narrative did the researcher ask additional questions, asking for clarification and detail. The interviews were recorded and transcribed for further analysis. For data analysis, key ideas from interview transcripts were identified during pre-coding. We used data driven**,** in-vivo coding to create categories and prepare higher order categorisation.

The limitations of the presented study are mainly based on the specificity of the chosen method. As narratives reveal individual experiences, they can show individuals’ identities, self-concepts, likes and dislikes of a person. Especially if the subject of the study is triggering strong emotions, as in this case. Another limitation lies in the way the research was conducted. Due to the pandemic situation, interviews were conducted using ICT tools, which could have disturbed the important intimacy between interviewer and interviewee and somehow affected the results.

With all these limitations in mind, procedures were put in place to achieve the highest possible trustworthiness of presented research. The trustworthiness of a study is a central aspect of the issues, referred to as validity and reliability, which were implemented from quantitative research. “Unlike validity in quantitative studies, qualitative validity is not a watertight product or a set of measures that can ensure the validity of the research” (Hayashi et al., 2019, p. 99). Validity in qualitative research is about the appropriate tools and data: whether the research question is valid for the desired finding, the choice of methodology is appropriate to answer the research question, the design is valid for the methodology, data analysis is adequate, and eventually the findings and conclusions are relevant to the context.

In order to achieve the required validity and reliability, the following measures were implemented:

- previous immersion in the field,
- theoretical triangulation,
- well-founded choice of adequate methodology and selecting relevant research techniques,
- precise description of the methodology and techniques of the study,
- conducting the study precisely,
- collecting data in a proper way, including adherence to the rules of interviewing, impartiality of questions and lack of suggestions, as well as ensuring adequate time and emotional distance,
- protecting the anonymity of interviewees in order to safeguard the freedom of conversation,
- aiming for a “wealth” of data and a diversity of sources,
- selecting suitably informed interviewees who are well-informed and willing to share their knowledge,
- ensuring that the questions asked are genuinely relevant to what is being the subject of the study,
- using appropriate language,
- reflection on informants’ feedback,
- high concern for accuracy in data analysis, especially in the context of coding and categorisation,
- researcher triangulation,
- comparison and reflection through the whole process,
- constant formative check of reliability.

As noticed, “in qualitative studies, validity cannot be seen as a product or something isolated. There are no protective measures. It is an ongoing process and should be confronted from the beginning of the research until its publication. Consequently, there is no single validity test although there are guidelines that allow a certain quality to be guaranteed to qualitative studies” (Hayashi et al., 2019, p. 103). Moreover, as Golafshani (2003) remarked, “It seems when quantitative researchers speak of research validity and reliability, they are usually referring to a research that is credible while the credibility of a qualitative research depends on the ability and effort of the researcher” (p. 600).

## Findings

Undoubtedly, information and communication technologies are an important tool in the work of teachers, irrespective of the type of institution in which they are employed. In the interviews of the researched group one can notice a high technological awareness, which was instilled already at the stage of training.

*In my previous studies, in my bachelor's programme, there were a lot of courses about teaching... using technologies in foreign language teaching, but they were mainly such subjects, in terms of classes, creating an application, making a video, showing different forms of presentations, something other than PowerPoint.* [T11, private school]

The interviewed English language teachers had previously used a variety of tools as part of their classroom teaching, regardless of the type of institution where they conducted their classes. In addition to using simple didactic materials (e.g. visual or audio) and various tools for activating participants, they also used technologically advanced devices. These included the digital versions of the coursebooks, which were offered by the publishers free of charge for the teachers during the first schools closure, but there were also other types of interactive platforms with materials and apps offered to language teachers, exceptionally, for free (MEiN, 2020).

*I have this version for an interactive whiteboard. So just like in class I use the interactive whiteboard, I have dropdown answers, menus, some puzzles etc.* [T1, public school]

*I am great, I am such a hacker that I can find (online) materials, books, everything.* [T9, language school]

Nevertheless, it should be stressed that their previous experience did not include conducting online synchronous classes with groups of younger children. Interviewees used the above-mentioned ICT tools mostly during regular school classes.

*I will start by saying that although I have a lot of teaching experience, I had never taught online.* [T9, language school]

*I have taught classes like this before, but not at this level and not with the formats that at least seemed new, like Zoom or Teams.* [T4, language school]

Educators had to confront a redefinition of the role of a teacher who usually met students in the classroom. Most importantly, the majority of respondents indicated that despite having at least basic skills in using ICT during classes, e.g. playing songs from YouTube or playing games on an interactive whiteboard, they were not technologically prepared for the transition from in-class technology-rooted instruction to distance online teaching. Therefore, lack of experience in this regard seems to be one of the reasons for the initial fear.

*Generally speaking, I was scared and unprepared at first, of course, like everyone else. And I could not at all imagine that our students would be able to participate in online lessons.* [T10, private school]

In view of the implementation of effective educational measures in the pandemic era, it seems extremely important to create optimal conditions for teachers to effectively adapt to new ways of teaching. Indeed, the transition to distance learning is a situation that requires preparation, whether technological, pedagogical or psychological.

### Implementation of ICT during the first wave of the COVID-19 pandemic in Poland

When describing the introduction of remote English language teaching in different types of educational institutions, it is important to highlight the differences in the approaches to language teaching and the use of ICT in different types of settings. The emphasis on the delivery of learning content may be weaker in those institutions where English is not the main subject and the primary issue is the delivery of a different range of material. This, but also other issues, such as the skills of the staff, top-down guidelines or the organisational skills of the management, determined how the implementation of distance education went and what form of working with children was eventually undertaken. In non-public institutions placing a strong emphasis on language education and often teaching other subjects in English, as well as in language schools, the transition to online classes with the use of such ICT tools as Microsoft Teams and Zoom was necessary.

*In our case it was different (than in ordinary schools), you either teach or you don't earn, just like that. And we had to do something.* [T9, language school]

However, public settings did not necessarily require teachers to teach in a synchronous way. Most teachers were left to make their own decisions on which ICT tools (e.g. email, electronic logbook, Linoit, Google Classroom) to use in contact with the students.

*We did not have a top-down order to conduct real-time lessons with such young children (...) well, English is not a core subject in grades 1*–*3.* [T6, public school]

*It was said that these lessons on Teams were kind of an option, they were not a requirement.* [T2, public school]

The general pattern of switching to distance education with the use of different ICT tools seems to be similar in all types of institutions. It can be divided into three stages:

1. asynchronous teaching, i.e., sharing materials, handouts, instructions, worksheets via the school’s website, email, electronic logbook or selected instant messaging services e.g., Facebook Messenger;
2. workshop development, consultations, tool searching, which led to teachers creating and sharing more technologically advanced materials: e.g., recordings, exercises using online applications and tools;
3. synchronous teaching, i.e., online classes conducted in real time using ICT tools, e.g. Microsoft Teams, Zoom.

While some schools only used asynchronous teaching, sharing materials without real time communication with students, in the case of the settings that undertook synchronous work, stage three followed. However, in various places the introduction of synchronous work happened at a different pace in different schools. One participant, a teacher working in a private primary school, described the implementation of distance learning in these words:

*For us, the way it worked was that at the beginning we had activities in the form of sample games. (...) And there were 5*–*6 sample games so that parents could play with their children. Then we started making videos. And at first there were two videos a week, and the rest of the time there were activities in the form of these games, which we could pass on to parents so that they could use them. Later, I think it was extended to four videos, and then there were online classes, we simply connected via Microsoft Teams.* [T12, private school]

It needs to be pointed out that the teachers themselves acknowledged the disparity in the introduction of remote teaching in different types of institutions.

*We talked to our friends who sent their children to a private school. And in the private school there are generally fewer children than there are teachers in our school, and they said that they switched to Zoom very quickly and had exactly the same number of remote lessons as they’d had before the whole lockdown.* [T2, public school]

The differences between the three types of institutions are noticeable in the dynamics and the process of introducing distance learning. In non-public institutions, the solutions were systemic and the instructions of working with the ICT tools were introduced quickly and efficiently.

*We used Microsoft Teams because our school has its own Microsoft tools, it has a Microsoft subscription. Everyone has some kind of Microsoft address out there, and it was easy for our IT people to make such groups on Teams.* [T10, private school]

Language schools implemented the chosen distance teaching solutions in consultation with parents, based on their own research into the ICT tools, and the process was quite fast.

*That was the time when we communicated with parents, we sent them information on how to use the Zoom, how to log in, etc., such detailed instructions. And that's how we were able to get it off the ground after only two weeks.* [T8, language school]

*Actually, in the first week of school closure there were no lessons in our school and we just sent some materials for kids to do, without any instructions, some kind of easy worksheets, but that only lasted the first week. And the online lessons, in real time, were introduced to us in the third week.* [T9, language school]

Public institutions, being dependent on external guidelines, and also the largest, experienced some organisational difficulties. Teachers did not always have information on how the classes should be conducted, or how to use different ICT tools, due to the lack of guidelines provided to schools by the authorities.

*And so I was wondering that in our public schools, even if we wanted to, it wouldn't be possible to carry it out in such a way, because in all the communications, in all the regulations from the Ministry, it was emphasised that you have to take care of children's work hygiene, that they can't sit so long at the computer, and so on. So even if we wanted to spend as much time sitting at computers and teaching children, we weren’t able to do it. Because it would be too exhausting for the children.* [T2, public school]

Due to the greater independence of non-public and language schools, combined with their lesser dependence on top-down guidelines, the performance of these types of institutions varied significantly.

In public schools, the transition to online synchronous teaching, if any, took several weeks. It is clear from the interviews that the reason for this was procedural issues, lack of guidance, and lack of awareness of the ICT tools available.

*It took the management a long time to find the right option for us. It was simply a top-down decision (...) Or maybe we began in April? Not right away, absolutely.* [T1, public school]

*The Teams came very late. For one thing, due to the fact that the management didn't know how and what to do either.* [T2, public school]

Non-public educational institutions and language schools were much quicker to adapt to the relevant regulations and restrictions. Despite initial ignorance of the uncertainties resulting from the lack of guidelines, after a couple of weeks teachers were conducting real-time lessons remotely, usually via Zoom or Microsoft Teams.

*It took two weeks* – *from the moment the schools closed and we stopped running classes, so it was those two weeks and it was a very intensive time for us, within the network and within my school of course, where we had to prepare ourselves and the equipment and the materials that we were going to use, so in general after those two weeks we were ready to go. So as we got closed this 13 March, we launched our first online lessons on 25 March.* [T8, language school]

Acquisition of technical literacy of ICT tools is another interesting issue. It mostly took place with the use of ICT tools. Teachers participated in training sessions, webinars and they also trained each other. However, we can see some differences in the organisation of further training between the different types of institutions. For example, teachers working in public schools were usually provided with links to instruction and training.

*There was no such formal training typical, but we got some links from the management. And that was it. There were some webinars and we were also enrolled.* [T1, public school]

*No, we were left to ourselves, as much as we could organise through some courses or our own activities, we had it organised. This is what it looked like.* [T3, public school]

Furthermore, in public schools, the instructions on how to use the video conferencing platforms were prepared by IT teachers for other staff members:

*The IT teachers were making instructions about Teams. Such tiny steps, day by day a new batch of instructions.* [T2, public school]

Some schools, in addition to publicly available materials, used training portals which ran webinars to prepare teachers for remote working. The teachers’ accounts also included materials prepared by publishers.

*As an association we bought access to such a portal and we had all the training sort of for free already, and during the pandemic I used four or five [webinars] plus what was available there at... Oh dear, I don't know now whether it was Cambridge or ... One of the publishers…* [T3, public school]

Staff members of language schools looked for tools for their own work and improved their workshop using the courses available. One of the interviewees emphasised the importance of own work and the necessity to search for information on different ICT tools independently.

*And also working on my own, researching and solving the problems I encountered. I knew there was Zoom, but I learned it by myself. I connected with my daughter at home, on two computers, and I worked on it myself, I read.* [T5, language school]

The statements of some teachers also included references to previous work in this area and they highlighted the importance of technical and methodological training initiatives already known before the pandemic.

*I had a whole range of tools to teach online. Of course, I took care of that much earlier. I was kind of already going for that, so I, as a teacher, was prepared for it.* [T5, language school]

Acquiring so many new technical skills on their own required teachers to devote a lot of time and energy. Irrespective of the place of employment, teachers were forced to create their professional workshop anew. As one of the interviewees said:

*Surely some people had to be convinced for these technologies* – *they had to, they had no other choice.* [T2, public school]

### Use of ICT tools during the first wave of the COVID-19 pandemic

In considering the introduction and use of ICT during the first wave of the pandemic, the topic of the tools themselves cannot be overlooked. What types of tools did the researched group use? In the interviews, the teachers mentioned a variety of tools, which the authors classified based on two criteria: functionality and use over time. We distinguished four groups of tools used by the informants:

1. Asynchronous communication tools – school website, email, MS Teams, Google Classroom, electronic logbook (e.g., Librus, BlueBoard), Facebook Messenger, voicemail
2. Synchronous communication tools – telephone, and video conferencing software (Skype, Zoom, MS Teams, Messenger)
3. Synchronous teaching tools – digital coursebook, worksheets, interactive whiteboard, smartboard, Kahoot.com, Genial.ly, Quizlet.com, Quizizz.com, Wordwall.net
4. Asynchronous teaching tools – school website, digital coursebook, worksheets, Kahoot.com, Genial.ly, Linoit.com, YouTube.com, Loom.com, ClassDojo.com, Quizlet.com, Quizizz.com, Wordwall.net

Some of the above-mentioned tools allow for both synchronous and asynchronous teaching. Because of a lack of guidance, and also due to time pressure, the teachers reached at first for tools they were familiar with, often originally serving other purposes, such as social network instant messaging. In the next stages of the transition to remote teaching, after discovering new possibilities or receiving guidance on the institution’s choice of tool, most of the respondents started using other platforms.

Public schools, in which the transition to real-time remote teaching was not always required, used synchronous communication tools to a lesser extent.

Since a large group of teaching tools can be used in both synchronous and asynchronous delivery modes, their use in all three groups of institutions seems to depend on the teachers’ knowledge of the tools and technical skills, their opinion on the relevance of using the available tools and their involvement in the preparation of the classes. Undoubtedly, teachers were forced to change their approach to using teaching aids which were irreplaceable in gaining the attention of children working with a computer screen rather than with a ‘live’ teacher. What had previously worked well during the regular lessons – playing team games, handing out worksheets, bringing toys and giving them to the children to play with became impossible during online distance education. However, during the remote classes the use of teaching aids still took place, though in a slightly altered form. Distance education pushed teachers working with groups to look for new ways of improving the lessons, and communicating with students via the Internet made it possible for educators to use interactive materials. Teachers participating in the study pointed out the opportunities they had discovered with digital technologies. They started using materials available on the Internet more often or creating materials which were suitable both for the students’ needs and the discussed topics.

### Other ICT uses

In the times of the pandemic, ICT have found their use in education, and not only when it comes to teaching children and young people. According to the research presented in this article, the use of ICT for training, self-help and support tasks for teaching staff was of particular importance. The interviewees mentioned available training courses and webinars as a predominant form of acquiring computing skills.

In the interviews teachers also indicated many forms of support they encountered when facing the challenge of the new reality in language education. Due to the difficulties such as a lack of information and guidance, Facebook was one of the places they found help and inspiration for classes, and read about how fellow professionals were doing. Teachers found there tips on the use of specific tools as well as ready-made teaching materials shared by others. Such informal channels of communication were an important part of support, as well as a place for the acquisition and improvement of skills.

*During this whole lockdown I signed up to so many websites and it took me through so many different tools and so much of everything, because really everyone was recommending everything. I found so much help from a teachers’ group on Facebook, because they recommended so many tools.* [T9, language school]

*We also shared materials with each other* – *teachers from different institutions. I think we posted it in a Facebook group* – *whoever wanted to share, whoever could, posted it. We shared materials, cool websites, and games. That’s also nice, because one could catch some inspiration.* [T12, private school]

Support with the use of ICT in the informants’ workplaces was at times more formal. The educational institutions themselves organised teachers’ self-help groups or methodological support.

*And, in addition, we had weekly staff meetings on (...) Microsoft Teams, where we exchanged experiences, some feedback, shared with the coordinators, who had a bit more influence on parents and wrote to them if anything happened, which had even bigger impact.* [T10, private school]

*Yes, I work in a preschool that is part of a chain (...). And there is a methodologist all the instructors report to, including me. And he was available for us every day at 8pm. (...) There was support.* [T12, private school]

Some institutions tried to provide other types of support, which is also another example of the use of modern technology. One interviewee mentioned a meeting with a psychologist cooperating with the school where the teacher is employed:

*We also had a Zoom meeting with a psychologist, and it was a psychologist who works with our school, where she just wanted to know what kind of problems we have, whether with the children or ourselves. And she was helpful. (...) and if someone would have liked to arrange an individual meeting with her later, this was also an option.* [T10, private school]

The role of IT departments or computing education teachers employed at the institutions where the study participants worked cannot be undermined. Their work, especially in the first two stages of introducing distance learning, seemed invaluable. The interviewees mentioned the IT teachers’ involvement in the selection of tools to be used by the school, the preparation of instructions or the technical handling of the delivery of materials during asynchronous teaching:

*I think computing education teachers in particular were overloaded with work. (...) One colleague is responsible for the website. And now all the material, from all the classes, had to be sent to her. She had to collect it, put it on the website...* [T1, public school]

The transition to remote teaching, in each of the forms mentioned above, resulted in the need for teachers to use ICT more extensively than before. Thus, certain categories of difficulties generated by the situation appeared in the interviews. The challenges the teachers faced are described in the following section.

### Challenges

By definition, modern technologies are designed to make things easier, solve problems and save time. And indeed, almost all teachers mentioned the fact of saving the time they used to spend on travelling to work and breaks between classes, which they had to spend on duty, keeping an eye on the children running around the school corridor. On the other hand, one of the biggest problems the teachers faced was the drastically increased length of class preparation time. While teachers had many ready-made aids for the regular lessons, such as textbooks, worksheets, games, puppets, etc., preparing new aids and using tools that they were not always familiar with, required a lot of time and effort from them. One of the participants said:

*Normally during the school year I try to do as little as possible at home, I practically don’t bring my pupils’ homework. (...) But during lockdown it was not possible and the work stretched over the whole day until late in the evening. When I tried to calculate how much I work a day, it sometimes turned out to be a dozen or so hours, instead of the 6*–*7 hours I normally have.* [T10, private school]

One more challenge identified by the informants was the children’s limited computer skills. It was simply impossible to complete the activities without the help of a parent or a guardian – an adult had to log on to the class for the child to be able to participate in activities. As one of the participants noticed, “*Certainly the support of parents was very important.”* [T8, language school].

A further challenge teachers faced was using the tools in a way which would facilitate carrying out activities in accordance with the methodology of teaching young learners so that the youngest children would be able to perform the tasks using the ICT. It needs to be underlined that in the youngest group, the assistance of parents was necessary for the delivery of the remote education programme.

On more than one occasion this presented a difficulty that teachers were unable to overcome. One of the study participants told us the parents were reluctant to help their own children learn English by means of ICT:

*And only a few 4- and 5-year-olds attended classes, because their parents said that they were too young and that they wouldn't, that they didn't know how to work with them.* [T1, public school]

Another issue was the constraints resulting from the specificities of working with young children. Until now, teachers have relied on ICT tools to support classroom activities, but it has been a challenge for them to use real-time teaching platforms, or to prepare materials that are attractive and simple enough for the youngest children to cope with. Many of them emphasised the difficulty of not engaging children in terms of physical activity, which, according to the methodology of teaching young learners, is characteristic for teaching this age group.

*The uncool thing about these classes is that I feel like it's impossible to do too many movement-based activities. The focus is rather on activities where you sit, you don't move much.* [T12, private school]

Teachers also pointed out as problematic the technical issues concerning the functions of the applications they had been using. These included, for example, children’s access to certain functions that teachers felt should not be available to them, or the ability to see a particular number of users at the same time.

*When we got connected, you couldn’t see all the children, I only saw four on the screen and not the others, which was a big difficulty (...) and another thing was that the kids were able to turn off my microphone. There were kids who already knew how to do this, and if they didn’t turn off my microphone, they did it to other kids. (...) I don't understand how participants can have this option to switch off somebody else’s microphone. Frankly, I do not know what purpose this would serve. If someone is giving a speech or holds a training session, others should not be able to switch off their microphone.* [T12, private school]

New, attractive forms of communication between the teacher and the class were also sometimes a field of experimentation for pupils who, having such a technical possibility, hindered the teaching in various ways.

Teachers also reported technical difficulties in preparing the teaching materials and instructions needed for asynchronous teaching.

*At times it looked like a film was being recorded, this red light was blinking all the time, and then when I pressed "save" and played it back to see how it came out, it turned out that only the first minute got recorded. It was really frustrating.* [T10, private school]

A few informants made comments about some improvement that they felt would make their work easier. One interviewee contended that notifications would have been helpful:

*I missed something like notifications (...) I had to check every now and then.* [T1, public school]

The technical problems mentioned by the study participants affected both teachers and students.

*It was such a technical difficulty for me. And I also knew that (...) not everything is displayed. Well, these were things I had no control over. Such technicalities. And I know that for children too I knew that sometimes they have technical problems, that sometimes they can't see or hear. Sometimes they get logged out of the lesson...* [T11, private school]

There were many factors influencing the quality of children’s remote learning. Among them, interviewees mentioned the living situation, which determines the workspace of both teachers and students. There were numerous statements in the interviews about the difficulties of working from home with other household members present at the same time, who also were busy with their own remote education or work.

*The biggest difficulty, which is not entirely under my control but we are working on it, is organising the right space for the children at home. What was a downside was noises coming from the other room or the parents watching over the child’s shoulder.* [T5, language school]

The sharing of space for educational and professional purposes often entailed the need to readapt the space, but this may have meant a deterioration in work comfort.

*I gave up the desk to my child, and apart from that I have a table, I could sit there, but also someone else could use it, so even logistically it was difficult to find a place to conduct classes.* [T4, language school]

Furthermore, the difficulties arising from the lack of or limited access to technical support of certain groups should also be highlighted. These difficulties affected both teachers and pupils. Preparing remote classes, whether synchronous or asynchronous, requires both parties to have devices and a stable Internet connection to communicate. The available data on Internet access in households do not appear to paint a whole picture in terms of the learning and teaching opportunities. In addition, the quality of the Internet connection also often seemed to be problematic. As one of the informants said:

*It’s also possible that the WiFi connection had something to do with it, because I hadn't used my connection that much before, as I was leaving for work. And I noticed that it’s pretty obvious that it has some limits. I saw that it was as if I was 'running out' of the Internet.* [T4, language school]

Another informant pointed to the fact that not all students have regular access to the Internet:

*Even if there is some Internet at home, it is not a permanent access, but for example at certain times. And most frequently these are parents' mobile phones rather than laptops. (...) We saw each other or could talk for a while, but for example, I couldn't share the screen so that we would do tasks together. So that was a huge negative, and unfortunately since March, in those 3.5 months, not much has changed.* [T3, public school]

A further concern expressed by the teachers in the interviews, mainly regarding households with more children, was the number and technical condition of the equipment available. Sometimes children could not participate in online classes because the devices were needed by their older siblings or were not available at all.

*The parents also said that they [children] don't need it, because if they have siblings attending school who have lessons, it's clear that the activities at school will be more important for the children and the parents rather than the kindergarten, right?* [T12, private school]

The interviewees mentioned that measures were taken to enable children to participate in classes. One teacher said:

*The school tried to provide computers somehow, but the Internet was not so good.* [T3, public school]

Despite this effort, another teacher working in a public school mentioned the concerns of the school management and the possible reluctance of parents towards online classes in real time, precisely due to equipment deficits and poor internet connection of the students’ families:

*The headmasters were afraid that parents would get disapproving if these [online] lessons were required, that there would be some kind of resentment, that "oh, because I have several children and only one computer", "oh, because we don't have the Internet" and so on…* [T2, public school]

One might guess that, especially in public schools, this was a significant constraint on the transition from asynchronous to synchronous teaching.

## Discussion and conclusions

The closure of educational institutions during the first wave of the pandemic in Poland resulted in the need to take up the challenge of transforming face-to-face education into a remote one. Teachers working at public and non-public institutions as well as language schools, surprised and unprepared for the situation, had to find their own ways to adapt to the new educational conditions. The difficult situation in which teachers were put forced them to use different forms of ICT mentioned in this text on a scale they could not have imagined. The study provided a brief overview of this transformation from the perspective of English language teachers and showed the differences and similarities between the three types of institutions represented by the informants. They concerned the way of working in institutions, the transition to remote teaching, the preparation of teachers for remote work, the type of tools used, technological difficulties, other ways of using ICT and challenges of the situation.

The results of the presented research show that the level of teacher preparation for remote education was not sufficient and the implementation of remote learning caused a lot of effort and stress for them. While even before the pandemic there were attempts to bring formal and informal education into the postdigital world to some extent, it is hard to disagree with the concern about skills gaps in the use of distance learning platforms expressed by UNESCO (2020). According to this report, published in April 2020, almost two thirds of the countries had the knowledge that their teachers did not have the necessary skills to facilitate distance education.

The benefits of teachers acquiring new technical skills appear to be invaluable, especially in the Polish reality where, according to research, almost immediately before the pandemic as many as 50% of schools were not using digital technologies (Plebańska, 2017). In addition, teachers admitted that the forced transition to remote education fostered their technological competence (Jaskulska & Jankowiak, 2020a). It needs to be emphasised that Poland is no exception in terms of lack of previous experience with remote education. For example, a great majority of teachers in Norway and the US did not have any experience with online teaching prior to the crisis, either (Gudmundsdottir & Hathaway, 2020).

The study results show that one strong conclusion can be drawn: all the informants want to return to face-to-face teaching. Considering the overall experience of using online education during the COVID-19 pandemic, as many as 59% of teachers do not want to use online education in the future (Plebańska et al., 2020). However, this does not mean that they do not see the advantages of the experience. Teachers mentioned the perceived benefits of using multimedia in the classroom and stressed that there were some elements they would definitely like to use in classroom lessons in order to make them more varied and to increase the effectiveness of teaching. However, it should be emphasised, which is also confirmed by Polish researchers (Plebańska, 2017), that digital resources were often used in English lessons even before the pandemic. Digital education worked in support of traditional education.

Moreover, thanks to the pandemic, modern technologies have become not only a working tool, a teaching aid for the teacher, but also a tool for the student, which creates additional educational opportunities for the future. The wide variety and introduction of tools which were not known before could result in a transition from the passive use of technology, dominant in Polish schools (Plebańska, 2017), to project-based, interactive, exploratory methods. At the moment, however, this is yet to be applied in the Polish educational reality (Plebańska et al., 2020).

It can therefore be concluded that, despite the numerous difficulties, the use of ICT in teaching will increase in the nearest future, as declared by all teachers participating in the presented study. This is also confirmed (on a smaller scale) by the results of the research conducted by the staff of the Adam Mickiewicz University in Poznań. In light of that study, teachers intend to use the skills they have acquired to a lesser extent in the future (Jaskulska & Jankowiak, 2020b). However, it should be stressed that the participants of that study were teachers of different subjects, mostly working in public schools. Therefore, their approach may be related to a more difficult experience with remote education that the public schools teachers had during the first schools closure.

The fact that the implementation of remote education in public schools took a lot of time and generated numerous negative comments can be justified in many ways. However, it seems clear from the data collected that the main reason for that were the organisational difficulties – the lack of clear guidelines provided by the bodies running the institutions and, what follows, insufficient recommendations and messages from the Ministry of Education. As other sources confirm, Polish non-public schools, which are very often smaller and more autonomous than the public ones, made remote online education easy, as having the resources to deliver this kind of tuition they in fact moved to synchronous teaching almost immediately after schools closure (Our Kids, n.d.). So it seems that improving the quality of communication and access to information has been a key issue for schools in the emergency situation of the transition to remote education. Some studies even suggest the imposition of tools and the need for a single platform to reduce chaos and improve the remote teaching process (Buchner et al., 2020). It would seem advisable to implement good practices in institutions where the introduction of real-time web-based learning has been more difficult. Further training enhanced by the lessons learnt from the first wave of the pandemic, as well as the informal sharing of applied innovations, may certainly improve the transition to remote synchronous education and the whole remote education process in schools where it is still cumbersome.

The *Remote Teaching Survey – Readiness, Tools and Challenges* study showed that when collating results across all participants (from Spain, USA, UK, Germany, Croatia and Australia), the most widely used remote teaching tools were: Google Hangouts (48% of participants), Zoom (42%) and Google Classroom (23%). However, further analysis of the data reveals a very strong divergence by country/language (Lawrence, 2020). The Polish characteristic fits into the general trend. Teachers participating in the study presented in this article also indicated the above tools, classified by the authors as belonging to the *communication* category. According to a research report published in June 2020 at the University of Warsaw, 31% of teachers taught online using synchronous communication platforms such as MS Teams, Zoom, Google Hangouts and others (Plebańska et al., 2020). Consistency in research on communication tools is not sustained when it comes to the use of teaching tools. The English language teachers involved in the study presented a wide range of teaching tools used in remote teaching. They mentioned using a variety of tools, many of which were interactive. The report prepared by the University of Warsaw shows that teachers most often use videos (21%) and multimedia presentations (18%). Online exercises are used by 15%, digital textbooks – 14% and interactive quizzes – 13% of the surveyed teachers. Less than 10% use mobile learning apps, digital learning games – 7% or online experiments – 2% (Plebańska et al., 2020). This may support the thesis that language teachers use modern technologies in teaching more frequently and more efficiently than other groups of teachers. At the same time, concern about the superficial use of technological tools by teachers in general seems justified. According to the model of the remote education process presented by Plebańska et al. (2020) using interactive activities is necessary to consolidate the content.

The European Commission’s report on the impact of COVID-19 on education contains an interesting remark relating to the education of younger pupils: *“teachers’ preparedness and positive attitude are key elements for the success of online learning platforms. Online instructors should be able to compensate for the lack of physical presence by setting up a virtual environment where all participants feel comfortable and teachers can be easily accessed.”* (Di Pietro et al., 2020, p. 10). Without any doubts, a committed teacher is able to bridge the gap that is a consequence of remote education, but must be supported by effective and well-known tools. That is probably why, according to our research, the initial asynchronous communication frequently involved the use of tools that were already familiar to everyone, i.e., the telephone, email, electronic logbook or school website. In addition, using social media channels and mobile apps for this purpose was even officially advised by the government (Cyfryzacja KPRM, 2020). The results we presented indicate also the technical difficulties present not only at the stage of learning how to use the tools. They were a struggle that teachers often mentioned while describing their further experiences. Along with the stress accompanying all participants and the workload associated with the need to prepare materials for remote education, these technical difficulties, both on the part of teachers and pupils, were not conducive to providing the recommended enabling environment.

The challenges connected with the implementation and use of remote education often originated at home, where there were deficits in equipment, space or lack of network access. Unequal access to ICT infrastructure at home was a huge concern for most countries, according to a report published by UNESCO (2020). Although the statistics show that the majority of Polish families have access to broadband Internet (GUS, 2020b), it needs to be pointed out that the quality of the connection leaves room for improvement, especially when everybody is using the Internet at the same time. To support not only schools but also families in the difficult task of distance learning it is necessary to introduce top-down approaches.

There is no doubt that external factors such as conditions at home, school or the workplace have an impact on learning and performance. Since in the age of the pandemic homes started to function as workplaces, the space and conditions available have become extremely important. According to the PISA 2018 study (Ostrowska & Sitek, 2020), Polish 15-year-olds rarely indicated difficult learning conditions, especially at home. Less than 4% of them declared a lack of their own desk and access to a computer at home (only 1.5% of pupils do not have any computer at home, the same number of pupils do not have an Internet connection at home), and slightly more than 4% – a lack of a quiet place to study. Nearly 90% declare that they have their own bedroom. It can be assumed that the home conditions of pupils in Poland, as studied before the outbreak of the pandemic, are mostly conducive to learning (Ostrowska & Sitek, 2020). However, it is not clear whether the own room is not shared with siblings, which may affect the quality of distance learning, and whether the declared access to a computer refers to the own computer for each child. When remote education was introduced, over a third of parents struggled with providing every child with a device for online learning (Librus, 2020). This is confirmed by the results presented in this article, which proves that the learning conditions positively evaluated in the PISA studies are not necessarily sufficient for the implementation of simultaneous remote e-learning by a greater number of household members. This is important, because up to 36% of teachers indicate students’ lack of equipment as one of the main problems with remote education (Buchner et al., 2020).

English teachers’ experiences of introducing distance teaching to young learners tended to be difficult also for other reasons. Due to their still limited skills, the young students are a group educationally disadvantaged in remote education. Comprehensive work with the youngest children was even considered impossible at times. It is also a result of the specific methodological skills of English language teachers of young learners. With that in mind, it seems to be very important to reflect on both the way we teach and the tools we use. As far as the youngest children are concerned, it seems necessary to support them in the process of remote learning, e.g. through the development of dedicated programmes or increased emphasis on the effectiveness and relevance of the provision of IT content in pre-school and early childhood education, to help children acquire necessary computing skills. Unquestionably “*it is not easy for educators working remotely to replicate the in-person classroom experiences that encourage play, autonomy, and experimentation in our youngest learners*” (Ostroff, 2020, p. 21). Hence, another recommendation is to disseminate good educational practices among teachers. On the other hand it seems to be crucial to enable parents to support remote education, especially in the case of the youngest children, for example by making their working hours more flexible or by hybridising their employment in order to ensure their participation in language education, from which they have sometimes been excluded.

It should be emphasised that, in relation to the current situation of the evolving pandemic, supporting teachers in the difficult process of implementing remote education and popularising synchronous teaching is still a valid topic. Research conducted at the Adam Mickiewicz University in Poznań shows that up to 35% of teachers declare that they feel supported by the school management, but almost 25% rated school management’s support as one of the worst elements of the functioning of the school during the period of remote education (Jaskulska & Jankowiak, 2020a). This information supports the results presented in this article, according to which negative evaluation of this support was expressed mainly by public school employees. At the same time, respondents stressed the great importance of self-help groups booming on social networks. As Kloskowska (2020) notes, remote education has further reduced the opportunities to communicate with other teachers and loosened deep social ties, thus contributing to feelings of lack of belonging. Therefore, online group support seems to have been extremely valuable in such difficult circumstances of isolation and stress. The support often involved activities that were not only of a psychological nature but also had a training character. It needs to be highlighted that according to the teachers, the most frequently used information source during the implementation of distance learning was not official training or instructions but the Internet (26%), followed by self-study (22%) and information provided by other teachers (21%) (Plebańska et al., 2020). This is confirmed by the results presented in this text. Therefore, it is worth taking further steps to improve the teachers’ workshop by developing training networks, tutorials, webinars and promoting the use of ICT in education, not only in the context of language education and working with young children. With the perspective of further lockdowns, it is worth using the insights from the first one, and taking action to eliminate the shortcomings of remote education.

## Future research

The problems addressed in this article are complex and multifaceted in nature. Due to this fact, it is advisable that future research should examine, among others, such areas as the state of remote education during further stages of the pandemic in Polish schools or differences in perception of remote teaching and ICT use across teachers of various subjects and age groups.

The next step will most likely be a larger-scale study designed to gather teachers’ ICT use experiences throughout the pandemic, with a special focus on effective remote working methods with the youngest pupils.

## Data Availability

The data that support the findings of this study are available from the corresponding author, upon reasonable request.

## Declarations

Additional declarations for articles in life science journals that report the results of studies involving humans and/or animals.
